# Analysis of a child who developed abnormal neuropsychiatric symptoms after administration of oseltamivir: a case report

**DOI:** 10.1186/s12883-015-0393-2

**Published:** 2015-08-05

**Authors:** Kaori Morimoto, Kei Nagaoka, Akira Nagai, Hirofumi Kashii, Masakiyo Hosokawa, Yukitoshi Takahashi, Takuo Ogihara, Masaya Kubota

**Affiliations:** Department of Pharmacology, Graduate School, Takasaki University of Health and Welfare, 60 Nakaorui-machi, Takasaki-city, Gunma Japan; Department of Drug Absorption and Pharmacokinetics, Tohoku Pharmaceutical University, 4-4-1 Komatsushima, Aoba-ku, Sendai-city, Miyagi Japan; Department of General Pediatrics and Interdisciplinary Medicine, National Center for Child Health and Development, 2-10-1 Okura, Setagaya-ku, Tokyo 157-8535 Japan; Division of Neurology, National Center for Child Health and Development, 2-10-1 Okura, Setagaya-ku, Tokyo 157-8535 Japan; Faculty of Pharmaceutical Sciences, Chiba Institute of Science, 15-8 Shiomi-cho, Choshi-city, Chiba Japan; Department of Pediatrics, National Epilepsy Center, Shizuoka Institute of Epilepsy and Neurological Disorders, Urushiyama 886, Aoi-ku, Shizuoka 420-8688 Japan

**Keywords:** Oseltamivir, Neuropsychiatric symptoms, Serum concentration

## Abstract

**Background:**

Neuropsychiatric side effects of oseltamivir occur occasionally, especially in infants and young patients, but nothing is known about possible contributory factors.

**Case presentation:**

We report a case of a 15-year-old Japanese female with influenza infection who developed abnormal psychiatric symptoms after administration of standard doses of oseltamivir. She had no history of neurological illness, had never previously taken oseltamivir, and had not developed psychiatric reactions during previous influenza infection. Her delirium-like symptoms, including insomnia, visual hallucinations, and a long-term memory deficit, disappeared after cessation of oseltamivir and administration of benzodiazepine. Detailed assessment was performed, including neurological examination (electroencephalogram, brain magnetic resonance imaging, single photon emission computed tomography with 99mTc-ethyl cysteinate dimer and with ^123^I-iomazenil, cerebrospinal fluid analysis and glutamate receptor autoantibodies), drug level determination and simulation, and genetic assessment (*OAT1, OAT3, CES1, Neu2*).

**Conclusions:**

Abnormal slowing in the electroencephalogram, which is characteristic of influenza-associated encephalopathy, was not observed in repeated recordings. The serum level determination of active metabolite Ro 64-0802 determined at 154 h after final dosing of oseltamivir was higher than the expected value, suggesting delayed elimination of Ro 64-0802. Thus, abnormal exposure to Ro 64-0802 might have contributed, at least in part, to the development of neuropsychiatric symptoms in this patient. The score on Naranjo’s adverse drug reaction probability scale was 6. Mutation of c.122G > A (R41Q) in the sialidase *Neu2* gene, increased CSF glutamate receptor autoantibodies, and limbic GABAergic dysfunction indicated by SPECT with ^123^I-iomazenil were found as possible contributory factors to the CNS side effects.

**Electronic supplementary material:**

The online version of this article (doi:10.1186/s12883-015-0393-2) contains supplementary material, which is available to authorized users.

## Background

Oseltamivir is a prodrug of the neuraminidase inhibitor [3R,4R,5S]-4-acetamido-5-amino-3-(1-ethylpropoxy)-1-cyclohexene-1-carboxylic acid (Ro 64-0802), which targets the influenza virus. Despite good tolerance of this drug by most patients, there have been reports of neuropsychiatric side effects, especially in infants and young patients [1]. However, contributory factors to the development of neuropsychiatric reactions after administration of oseltamivir are presently unknown.

## Case presentation

**Fig. 1:**
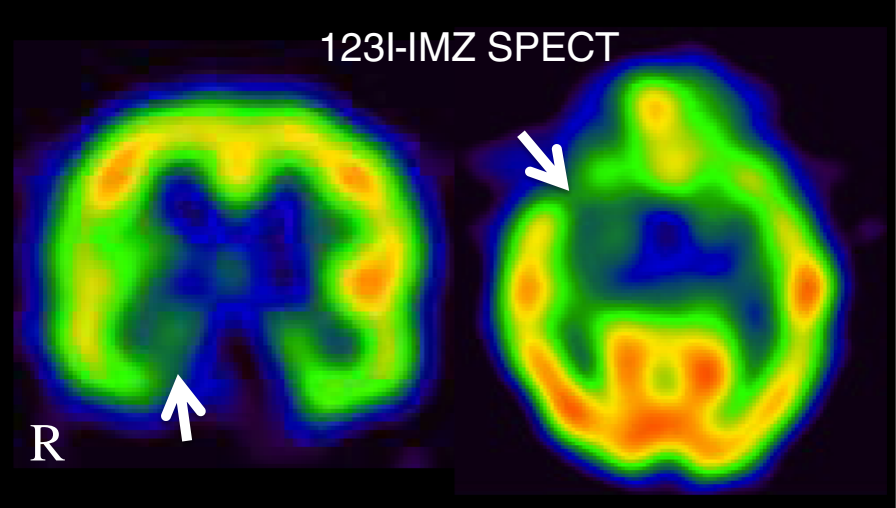
SPECT with ^123^I-iomazenil 10 days after admission revealed decreased benzodiazepine receptor binding in the right medial temporal area (*arrows*)

This previously healthy 15-year-old Japanese girl developed fever and was diagnosed with influenza A virus infection based on a positive result with a rapid detection kit in 2009, during the pandemic influenza (H1N1) period. She had not previously taken oseltamivir, and had not developed neuropsychiatric symptoms in her previous influenza infection; this was her first admission to a neurological ward. The day after oseltamivir was started, her fever subsided, but the family noticed unusual behavior, such as doing her makeup at midnight and littering her room. She took 5 doses of 2 mg/kg (150 mg/day, b.i.d., 36 kg in weight) of oseltamivir every twelve hours and concomitantly took dihydrocodeine, ambroxol, benproperine, azithromycin and clarithromycin. There is no report of neuropsychiatric symptoms associated with these concomitant medications, except clarithromycin (clarithromycin occasionally induces altered mental status accompanied with electroencephalogram (EEG) abnormality, mostly in adults with psychiatric illness [2]). The symptoms did not fluctuate according to oseltamivir dosing times. After 3 days, she suddenly left her home and was found standing in front of an unknown apartment. Her family discontinued oseltamivir at that point. Because her bizarre behaviors continued after cessation of oseltamivir (for example, she complained of hearing voices of strange men), she was admitted to our hospital with suspected influenza encephalopathy. She developed delirium-like symptoms, such as insomnia, disorientation, visual hallucinations, a long-term memory deficit, abnormal behaviors (*e.g.*, persistent ritual sneezing) and delusion (*e.g.*, insisting that she was pregnant), but showed normal responses intermittently, especially during benzodiazepine infusion (diazepam or midazolam). Midazolam infusion (1 mg) enabled her to give correct verbal responses to questions about her name, her friends’ names, her age, and her address, as well as to perform mental calculations (additions and subtractions). Seizures were not observed and abnormal slowing in the EEG, which is characteristic of influenza-associated encephalopathy, was not observed in repeated recordings. Cerebrospinal fluid (CSF) study and repeated brain MRIs showed no abnormality. SPECT with ^99m^Tc-ethyl cysteinate dimer showed no abnormal cerebral perfusion, while SPECT with ^123^I-iomazenil (performed 3 days after discontinuation of benzodiazepine) showed decreased benzodiazepine receptor binding in the right medial temporal area (Fig. 1).

Glutamate receptor autoantibodies (GluRAbs), including GluRε2-NT2, GluRε2-CT1, GluRδ2-NT and GluRδ2-CT, were within normal limits in serum, but were increased in CSF (Additional file 1: Table S1).

Finally, she completely recovered within 2 weeks after cessation of oseltamivir and administration of benzodiazepine (oral nitrazepam or lorazepam, with continuous midazolam infusion for 5 days in the middle). Naranjo’s adverse drug reaction probability scale [3] gave a score of 6.

### Drug level determination and simulation

**Fig. 2:**
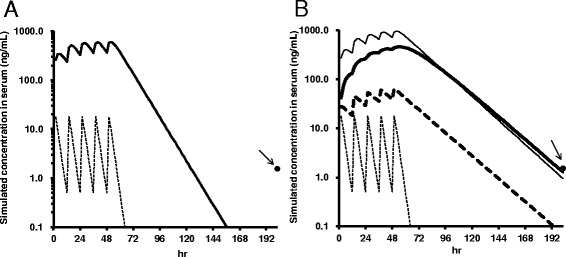
Simulations of serum concentrations of oseltamivir and Ro 64-0802. **a** Simulation with normal parameters. Closed circle (*arrow*), observed Ro 64-0802 concentration; dotted line, simulated oseltamivir concentration; solid line, simulated Ro 64-0802 concentration. **b** Simulations of Ro 64-0802 and oseltamivir concentrations. Closed circle (arrow), observed Ro 64-0802 concentration; dotted lines, oseltamivir; solid lines, Ro 64-0802. Thin lines indicate simulation results using an elimination rate of Ro 64-0802 of one-half of the normal value. Bold lines indicate simulation results using a conversion rate of oseltamivir to Ro 64-0802 of one-tenth of the normal value

At 154 h after final dosing of oseltamivir, blood and cerebrospinal fluid (CSF) were taken for drug level determination and genetic assessment. The serum level of oseltamivir was under the detection limit (0.5 ng/mL) and that of its active metabolite Ro 64-0802 was 1.30 ng/mL. CSF levels of oseltamivir and Ro 64-0802 were both under the detection limit (0.5 ng/mL). The observed serum level of Ro 64-0802 in this patient was higher than the expected level of Ro 64-0802 based on the pharmacokinetic parameters of age-matched healthy subjects, suggesting delayed excretion of Ro 64-0802 (Fig. 2a). The pharmacokinetics of oseltamivir in patients with influenza is qualitatively similar to that in healthy subjects [4].

Oseltamivir is exclusively converted by hepatic carboxylesterase CES1 to Ro 64-0802, which is excreted into urine by glomerular filtration and tubular secretion *via* organic anion transporter OAT1 and/or OAT3 [5, 6]. Delayed excretion of Ro 64-0802 could be due either to decreased conversion of oseltamivir to Ro 64-0802 or to decreased elimination of Ro 64-0802 itself. Simulation studies indicated that the observed serum concentration of Ro 64-0802 at 154 h after final dosing was consistent with a decrease to one-tenth of normal in the conversion rate of oseltamivir to Ro 64-0802 or with a decrease to one-half in the elimination rate of Ro 64-0802 (Fig. 2b, Additional file 2: Table S2 and Additional file 3: Table S3). The former case, but not the latter, would also result in increased concentrations of oseltamivir, though in both cases, the oseltamivir concentration would have been at an undetectable level by 154 h after the final dose. The patient’s renal function was normal. The patient was not receiving any drugs known to inhibit OAT1, OAT3 or CES1.

### Genetic assessment

Gene sequencing of *OAT1* and *OAT3* and diplotype analysis of *CES1* failed to explain her abnormal exposure to Ro 64-0802. Examination of the single nucleotide polymorphism of c.122G > A(R41Q) in the human sialidase *Neu2* gene showed that the patient was a heterozygous carrier of this variation [7].

## Discussion and conclusions

As far as we know, this is the first case report that includes the results of a comprehensive examination of the underlying mechanisms of psychiatric reactions associated with oseltamivir. The results of drug level determination and simulation suggested that enhanced exposure to either Ro 64-0802 alone or to both oseltamivir and Ro 64-0802 might have occurred in this case. Although influenza encephalopathy itself causes similar symptoms, and it was ethically unacceptable to examine the effect of re-administration of oseltamivir, this neuropsychiatric reaction appears to have been drug-induced, because EEG changes characteristic of influenza-associated encephalopathy was not observed and the symptoms disappeared after drug cessation. A previous report indicated that oseltamivir up to 450 mg twice daily is well-tolerated in healthy volunteers, and no drug-related psychiatric symptoms were observed [8]. However, the safety and pharmacokinetics of oseltamivir and Ro 64-0802 in subjects with severe influenza infection are presently unknown. In our patient, repeated measurements of the same samples and simulation studies clearly demonstrated enhanced exposure to Ro 64-0802, although the precise reason for this was not established. The brain concentration of oseltamivir and/or Ro 64-0802 is likely to be the key determinant of neuropsychiatric side effects. Although oseltamivir and Ro 64-0802 were not detected in CSF, it is considered that enhanced brain exposure to oseltamivir and/or Ro-64-0802 would have occurred, because brain-to-plasma ratios were reported to be linear up to 100 mg/kg (with values of 0.1 for oseltamivir and 0.005–0.02 for Ro 64-0802) in mice [9]. Efflux transport of oseltamivir is mediated by P-glycoprotein (ABCB1) at the blood–brain barrier, and therefore reduced P-glycoprotein activity due to factors such as genetic polymorphism [10] and inhibition by pro-inflammatory cytokines [11] or concomitant drugs could result in an increased brain concentration of oseltamivir. For example, our patient concomitantly received azithromycin, which may inhibit P-glycoprotein [12]. Although Ro 64-0802 is not a substrate of P-glycoprotein, animal studies indicate that it undergoes active efflux at the BBB mediated by transporter(s) such as *OAT3* [13]. Therefore, reduced efflux transport of Ro 64-0802 is another possibility, although no genetic abnormality of *OAT3* was found in our patient.

Based on the neuropsychiatric symptoms following influenza A infection and the increase in CSF GluRAb, our patient's clinical course could be categorized as non-herpetic limbic encephalitis [14], but the precise nosological position remains inconclusive. Our patient’s clinical course somewhat resembled that of autoimmune limbic encephalitis, but differed in disease duration (much longer in autoimmune limbic encephalitis), the efficacy of benzodiazepine, the absence of abnormal EEG and CSF findings, and the absence of seizures and movement disorders during the clinical course (seizures and movement disorders are often observed in patients with typical autoimmune limbic encephalitis according to Dalmau’s group [15, 16]). Further, our GluRAbs were different from Dalmau’s NR1/NR2 heteromers of *N*-methyl-Daspartate receptor (NMDAR), and the disease sensitivity of our GluRAbs appeared to be low.

Limbic GABAergic dysfunction might be involved in the pathogenesis, considering the transience of the symptoms and the later persistent efficacy of benzodiazepine in our patient. This idea is further supported by the ^123^I-IMZ SPECT findings, which suggested a decrease in the right medial temporal benzodiazepine/GABA-A receptors. Usami *et al.* [17] reported that oseltamivir and its active metabolite enhanced neuronal synchronization and induced population burst events of rat hippocampal CA3 networks in a concentration-dependent manner. They suggested that the oseltamivir-induced population bursts may be shaped by the phasic activity of inhibitory interneurons, rather than pyramidal cells. Thus, GABAergic inhibitory interneurons might be an action target of oseltamivir.

The genetic polymorphism of c.122G > A (R41Q) in the *Neu2* gene of our patient resides near the active site of this enzyme and has the effect of increasing the binding affinity of Ro 64-0802, making the enzyme more sensitive to inhibition by Ro 64-0802 *in vitro* [4]. We speculate that oseltamivir induced excessive population bursts, which were further enhanced by the *Neu2* gene SNP, resulting in down-regulation of GABAergic receptor, leading to the SPECT change. Because of the similarity between the neuropsychiatric reaction to oseltamivir and the symptoms of human sialidase-related disorders (epilepsy [18], seizures [19], mental deterioration [20], impaired intelligence [20]) and the role of sialidases in neuronal excitation [21, 22] and synaptic plasticity [23], we speculate that possession of this variant may play a role in the occurrence of severe adverse reactions to oseltamivir. Further clinical investigation will be needed to test this idea.

In conclusion, abnormal exposure to oseltamivir and/or Ro 64-0802 probably contributed to the development of neuropsychiatric symptoms in this patient, at least in part. Factors that might increase the risk of CNS side effects after administration of oseltamivir include *Neu2* mutation, exclusively observed in Asians, and increased CSF GluRAbs. GABAergic inhibitory neurons may play an important role in the pathogenesis of the psychiatric symptoms.

### Consent

Informed consent was obtained from the patient and her parents after oral or written explanation, according to the ethical principles of the Declaration of Helsinki. A copy of the written consent is available for review by the Editor of this journal.

## Additional files

Additional file 1: Table S1. Measurements of glutamate receptor autoantibodies (OD values with ELISA method) in our patient. (DOCX 29 kb) Additional file 2: Table S2. Simulated serum concentrations of oseltamivir and Ro 64-0802 in our patient. (DOCX 30 kb) Additional file 3: Table S3. Pharmacokinetic parameters used for the simulation of plasma concentration - time curve in our patient. (DOCX 30 kb)

## Abbreviations

EEG: Electroencephalogram
CSF: Cerebrospinal fluid
GluRAbs: Glutamate receptor autoantibody
OAT: Organic anion transporter
CES: Carboxylesterase
Neu2: Neuraminidase 2
