# Supplementary material for: Analysis of a child who developed abnormal neuropsychiatric symptoms after administration of oseltamivir: a case report
Source: BMC Neurol. 2015 Aug 5;15:130. doi: 10.1186/s12883-015-0393-2 (PMC4526296; doi:10.1186/s12883-015-0393-2)
Supplement: Additional file 1: Table S1. — Measurements of glutamate receptor autoantibodies (OD values with ELISA method) in our patient. (DOCX 29 kb) [file 12883_2015_393_MOESM1_ESM.docx]

Additional file 1: Table S1. Measurements of glutamate receptor autoantibodies (OD values with ELISA method) in our patient.

|  | GluRε2-NT2 | GluRε2-CT1 | GluRδ2-NT | GluRδ2-CT |
| --- | --- | --- | --- | --- |
| Serum (2 days after admission) | 0.504 | 0.537 | 0.489 | 0.537 |
| Serum (10 days after admission) | 0.542 | 0.528 | 0.557 | 0.541 |
| Serum, Control (n=53) | 0.523±0.233 | 0.556±0.140 | 0.641±0.230 | 0.765±0.429 |
| CSF (2 days after admission) | 0.952 | 1.07 | 1.122 | 1.032 |
| CSF, Disease control (non-inflammatory partial epilepsy n=45) | 0.216±0,083 | 0.230±0.091 | 0.274±0.147 | 0.316±0.171 |
