# Supplementary material for: Analysis of a child who developed abnormal neuropsychiatric symptoms after administration of oseltamivir: a case report
Source: BMC Neurol. 2015 Aug 5;15:130. doi: 10.1186/s12883-015-0393-2 (PMC4526296; doi:10.1186/s12883-015-0393-2)
Supplement: Additional file 2: Table S2. — Simulated serum concentrations of oseltamivir and Ro 64-0802 in our patient. (DOCX 30 kb) [file 12883_2015_393_MOESM2_ESM.docx]

Additional file 2: Table S2. Simulated serum concentrations of oseltamivir and Ro 64-0802 in our patient

|  |  |  | Serum concentration | | AUC |
| --- | --- | --- | --- | --- | --- |
|  |  |  | Cmax (5th) | 154 h after final dosing |  |
|  |  |  | ng/mL | ng/mL | ng・h/mL |
| Observed |  | Oseltamivir |  | n.d. |  |
|  |  | Ro 64-0802 |  | 1.30 |  |
| Simulation | Normal | Oseltamivir* | 18 | 0.00 | 354 |
|  |  | Ro 64-0802** | 608 | 0.00 | 30913 |
|  | 1/10 esterase activity | Oseltamivir | 64 | 0.07 | 3351 |
|  |  | Ro 64-0802 | 463 | 1.30 | 31188 |
|  | 1/2 urinary excretion of | Oseltamivir | 18 | 0.00 | 354 |
|  | oseltamivir carboxylate | Ro 64-0802 | 970 | 0.95 | 56536 |

n.d., not detected (< 0.97 g/mL); AUC, area under the serum concentration-time curve

*: Massarella JW, et al., *J Clin Pharmacol.*, 40, 836-843 (2000)

**: Basic product information of Tamiflu (13-18 years-old)
