# Supplementary material for: Analysis of a child who developed abnormal neuropsychiatric symptoms after administration of oseltamivir: a case report
Source: BMC Neurol. 2015 Aug 5;15:130. doi: 10.1186/s12883-015-0393-2 (PMC4526296; doi:10.1186/s12883-015-0393-2)
Supplement: Additional file 3: Table S3. — Pharmacokinetic parameters used for the simulation of plasma concentration - time curve in our patient. (DOCX 30 kb) [file 12883_2015_393_MOESM3_ESM.docx]

Additional file 3: Table S3. Pharmacokinetic parameters used for the simulation of plasma concentration - time curve in our patient

| Assumption |  | V/F | k01 | k10 |
| --- | --- | --- | --- | --- |
|  |  | mL/kg | hr-1 | hr-1 |
| Nomal parameters | Oseltamivir* | 3739 | 0.391 | 0.086 |
|  | Ro 64-0802** | 3739 | 0.391 | 0.086 |
| 1/10 esterase activity | Oseltamivir | 64895 | 1.647 | 0.045 |
|  | Ro 64-0802 | 3739 | 0.045 | 0.086 |
| 1/2 urinary excretion of | Oseltamivir | 64895 | 1.647 | 0.045 |
| Ro 64-0802 | Ro 64-0802 | 3739 | 0.391 | 0.047 |

V/F, distribution volume/absorption rate; k01, absorption rate constant; k10, elimination rate constant

*: Massarella JW, et al., *J Clin Pharmacol.*, 40, 836-843 (2000)

**: Basic product information of Tamiflu (13-18 years-old)
